# Editorial: Next-Generation Genetically-Encoded Fluorescent Sensors

**DOI:** 10.3389/fncel.2020.627792

**Published:** 2020-12-03

**Authors:** Shai Berlin, Elizabeth C. Carroll

**Affiliations:** ^1^Department of Neuroscience, The Ruth and Bruce Rappaport Faculty of Medicine, Technion Israel Institute of Technology, Haifa, Israel; ^2^Department of Imaging Physics, Delft University of Technology, Delft, Netherlands

**Keywords:** biosensor, fluorescent, genetically encoded activity indicators, photoactivable, neuron, activity, disease

Fluorescent probes, or biosensors, are cleverly designed molecules that are able to transform the act of binding/reacting with a unique target (e.g., analyte) into a fluorescent signal (ΔF/F). Of the various kinds (e.g., small molecules, fluorescent proteins), genetically-encoded fluorescent sensors are particularly popular as these can be targeted to select cells or subcellular regions via gene promoters, conditional expression systems, protein recombination schemes or localization sequences, to name a few. In particular, the field of Genetically-Encoded Indicators (GE*x*I; *x*— calcium, neurotransmitter, voltage, etc.) continues to flourish as researchers explore and develop new methods to improve sensitivity, specificity, and compatibility of probes for multi-channel microscopy. Contemporary GExIs include a single fluorescent protein as their fluorescent core (commonly GFP or RFP), making them smaller than FRET-based probes as well as allowing users to more easily combine these—crosstalk free—with added optogenetic or photochemical tools that require their own set of wavelengths for activation. Owing to these features, GExIs have become valuable tools for cell biologists with a broad range of questions. In this Research Topic on “Next Generation Genetically-Encoded Fluorescent sensors,” we collate reviews and research articles representative of the breadth of the field today, including descriptions of the functioning principles of genetically-encoded fluorescent biosensors, their potential and pitfalls, examples of applications, and original reports describing the development of new probes or use of novel biosensors to study diseases.

We present three retrospective articles by leading scientists in the field. The review by Leopold et al. provides a concise overview of current biosensors best suited for imaging neurotransmission and neuromodulation in neurons. The authors describe operating principles of how binding of target molecules changes the fluorescence of the probe, and detail a workflow for their development. Complementary to this, Ravotto et al., offer a fresh perspective on probes that link G-Protein Coupled Receptors to circularly-permuted GFP (cpGFP), and their potential for remote and real-time monitoring of various neurotransmitters, such as dopamine, in the brains of living animals. The authors discuss potential challenges when using these emerging tools and further suggest new designs. Finally, a comprehensive review by de Beer and Giepmans details the use of probes based on single-chain antigen binding proteins (nanobodies) for subcellular identification of endogenous proteins. The authors review the current state of the art of nanobodies fused to fluorophores and peroxidases, including discussion of different labeling strategies and applications for multi-modal nanobody probes in correlative light and electron microscopy.

Four articles in the collection focus on probing diseases by developing novel assays and adapting GExIs to meet a specific need. Waldeck-Weiermair et al., describe their efforts to understand sub-organellar Ca^2+^-uptake by mitochondria. To address this, the authors explore novel targeting peptides to localize extant green- and red-emitting GECIs (c-calcium) to the intermembrane space and cristae lumen of mitochondria. Their approach proved successful and they employ it to examine the effects of gene knock-down on Ca^2+^-dynamics in mitochondria. They go on to show that several GECIs of different colors can be combined in the same cell to examine Ca^2+^-levels in distinct sub-compartments of the organelle. Harlen et al., focus their attention on Ca^2+^-dynamics of the endoplasmic reticulum (ER). Here, the authors try to understand how different disease-relevant mutations in rhodopsin or α-synuclein affect cell stress and intrinsic ER-signaling. This goal motivated them to devise a semi-high throughput live-cell assay for ER-mediated cell stress and second messenger signaling. They analyze single-cell Ca^2+^-profiles by combining use of a green GECI, a red cAMP biosensor (R-cADDis) and an optical actuator bPAC (blue light-activated adenylyl cyclase). Together these provide new views on disease processes. Bera et al., also look to the ER, where instead of studying endogenous analytes within the organelle, they engineer a novel family of biosensors—iSKetSnFR (Intensity-based S-ketamine-sensing fluorescent reporters)—to examine whether S-ketamine, an antidepressant drug, can pass the plasma membrane and enter the ER lumen. The authors find that S-ketamine can rapidly enter and exit the ER, which leads them to propose that the antidepressant actions of the drug may also involve intracellular activity of the drug on organellar ion channels, receptors, and transporters. Das et al. also explore a disease of the central nervous system, namely multiple sclerosis (MS). They ask whether prolonged demyelination and remyelination have any effect on neuronal activity in the hippocampus. The authors combine electrophysiological recordings and imaging of GECIs to monitor neural firing during demyelination over a period of 100 days. They find that demyelination greatly reduces synaptic transmission and firing rates in CA1 and DG neurons *in vivo*. The authors suggest that GECIs can also be used to examine effects of therapeutics on CNS neurons.

Lastly, two original reports focus on the development of photoactivatable (PA) fluorescent probes. PA-probes are particularly useful when trying to obtain relevant fluorescent signals solely from cells defined by the user without excessive background fluorescence from nearby cells. Lee et al., engineer photoactivatable genetically encoded voltage- (GEVI) and pH (GEPI)-indicators and characterize these in HEK293 cells. They first explore several probe designs for a GEVI, namely using a single fluorescent protein (cpGFP), a FRET pair or a pH-sensitive variant of GFP, ecliptic pHluorin. The candidate bearing the ecliptic pHluorin exhibited both coveted features, explicitly photoactivation and voltage-dependent fluorescence changes. This led the team to the engineering of a pH-sensitive photoactivatable GFP that varies its brightness in response to different intracellular pH. Analogously, Hussein and Berlin explore the photoactivation of red GECIs, leading them to develop PA-R-GECO, a red photoactivatable Ca^2+^-probe. This expands the color palette of photoactivatable Ca^2+^-indicators. In the process of testing photoactivation in several other red GECIs, they also create a new photoactivatable RFP variant, namely PA-mRuby3, that undergoes strong photoactivation.

Collectively this collection of articles highlight the rich possibilities for discovery remaining in the field of genetically-encoded fluorescent sensors.

## Author Contributions

SB and EC wrote the manuscript. Both authors contributed to the article and approved the submitted version.

## Conflict of Interest

The authors declare that the research was conducted in the absence of any commercial or financial relationships that could be construed as a potential conflict of interest.

